# Geovisual analytics to enhance spatial scan statistic interpretation: an analysis of U.S. cervical cancer mortality

**DOI:** 10.1186/1476-072X-7-57

**Published:** 2008-11-07

**Authors:** Jin Chen, Robert E Roth, Adam T Naito, Eugene J Lengerich, Alan M MacEachren

**Affiliations:** 1 GeoVISTA Center, Department of Geography, the Pennsylvania State University, University Park, USA; 2 Department of Public Health Sciences, the Pennsylvania State University, Hershey, USA

## Abstract

**Background:**

Kulldorff's spatial scan statistic and its software implementation – SaTScan – are widely used for detecting and evaluating geographic clusters. However, two issues make using the method and interpreting its results non-trivial: (1) the method lacks cartographic support for understanding the clusters in geographic context and (2) results from the method are sensitive to parameter choices related to cluster scaling (abbreviated as scaling parameters), but the system provides no direct support for making these choices. We employ both established and novel geovisual analytics methods to address these issues and to enhance the interpretation of SaTScan results. We demonstrate our geovisual analytics approach in a case study analysis of cervical cancer mortality in the U.S.

**Results:**

We address the first issue by providing an interactive visual interface to support the interpretation of SaTScan results. Our research to address the second issue prompted a broader discussion about the sensitivity of SaTScan results to parameter choices. Sensitivity has two components: (1) the method can identify clusters that, while being statistically significant, have heterogeneous contents comprised of both high-risk and low-risk locations and (2) the method can identify clusters that are unstable in location and size as the spatial scan scaling parameter is varied. To investigate cluster result stability, we conducted multiple SaTScan runs with systematically selected parameters. The results, when scanning a large spatial dataset (e.g., U.S. data aggregated by county), demonstrate that no single spatial scan scaling value is known to be optimal to identify clusters that exist at different scales; instead, multiple scans that vary the parameters are necessary. We introduce a novel method of measuring and visualizing reliability that facilitates identification of homogeneous clusters that are stable across analysis scales. Finally, we propose a logical approach to proceed through the analysis of SaTScan results.

**Conclusion:**

The geovisual analytics approach described in this manuscript facilitates the interpretation of spatial cluster detection methods by providing cartographic representation of SaTScan results and by providing visualization methods and tools that support selection of SaTScan parameters. Our methods distinguish between heterogeneous and homogeneous clusters and assess the stability of clusters across analytic scales.

**Method:**

We analyzed the cervical cancer mortality data for the United States aggregated by county between 2000 and 2004. We ran SaTScan on the dataset fifty times with different parameter choices. Our geovisual analytics approach couples SaTScan with our visual analytic platform, allowing users to interactively explore and compare SaTScan results produced by different parameter choices. The Standardized Mortality Ratio and reliability scores are visualized for all the counties to identify stable, homogeneous clusters. We evaluated our analysis result by comparing it to that produced by other independent techniques including the Empirical Bayes Smoothing and Kafadar spatial smoother methods. The geovisual analytics approach introduced here is developed and implemented in our Java-based Visual Inquiry Toolkit.

## Background

### The Kulldorff spatial scan statistic and SaTScan

The scan statistic is a method for detecting non-random clustering in multi-dimensional point or near-point datasets [1]. Although there are numerous variants of the scan statistic, we focus upon the two-dimensional spatial scan statistic because of its potential for identifying geographic clusters of increased disease risk. The concept of a disease cluster can be defined as an unusually high concentration of disease events in a region unlikely to have occurred by chance [2,3]. Put simply, a disease cluster is any area within the study region of significant elevated risk for a disease [4], which is often referred to as a hot-spot cluster. One of the earliest discussions and implementations of the spatial scan statistic was in the Geographical Analysis Machine (GAM) by Openshaw and colleagues [5]. Of particular interest to the present research is the Kulldorff [6] spatial scan statistic because it is both deterministic (i.e., it identifies the locations of clustering) and inferential (i.e., it allows for hypothesis testing and evaluation of significance). Kulldorff's SaTScan [7] software is the most widely used implementation of the spatial scan statistic method. The research reported here specifically enhances interpretation of the output derived from SaTScan; and the methodology described in the research is applicable to other spatial scan statistic implementations (and other cluster detection methods) with minor alterations. The SaTScan implementation of the spatial scan statistic is capable of detecting both circular and elliptical clusters; for simplicity, we consider only circular clustering for the remainder of the paper, but our method is equally applicable to the elliptical variant.

The spatial scan statistic and the SaTScan software is described in detail by Kulldorff and colleagues in a series of papers [6,8-10]. It is summarized here to provide context for the geovisual analytics approach we introduce for enhancing interaction with and interpretation of SaTScan results. For disease mortality, the null hypothesis of the Kulldorff spatial scan statistic states that deaths are randomly distributed in geographic space and the expected death count is proportional to the population at risk (i.e., adjusted by age or other covariates). The alternative hypothesis is that there is increased mortality within an area as compared to the outside areas. The spatial scan statistic imposes a circular window on the map and moves the circle centre over each point location so that the window includes different sets of neighbouring points at different positions [10]. For enumerated data, an areal unit will be included if its centroid is within the bounds of the circle. By adjusting the centre location and radius, the method generates a large number of distinct circular windows, each including a different set of neighbouring points. At each point location, the radius of the circle is increased continuously from '0' to a user-defined maximum radius [10]. The user-defined maximum radius used by SaTScan is referred to subsequently as *maximum-size*. The maximum-size parameter sets an upper bound on the circle radius in one of two ways: (1) by specifying the maximum percentage of the total population at risk within the circle or (2) by specifying the geographic extent of the circle. The former is the default and is used in the research reported here. When setting the maximum-size based upon population at risk, the default maximum-size value, as recommended in the SaTScan user guide [11], is set to 50% of the total population at risk – with this setting, a reported cluster can contain at most 50% of the total population at risk.

SaTScan detects potential clusters by calculating a likelihood ratio for each circle. The likelihood ratio is proportional to Equation 1:

(1) $${\left(\frac{c}{e}\right)}^{c}{\left(\frac{C-c}{C-e}\right)}^{C-c}I()$$

where *C *is the total number of cases, *c *is the observed number of cases within a circle, and *e *is the adjusted expected number of cases within the circle. I() is a binary indicator that facilitates identification of high-risk clusters (hotspots), low-risk clusters ("coldspots"), and both. When SaTScan is set to scan for high-risk clusters, I() is equal to '1' when *c *> *e *and equal to '0' otherwise; for low-risk clusters, the ">" would change to "<"; and for both, I() = 1 [11]. The circle with the maximum likelihood ratio among all radius sizes at all possible point locations is considered as the most likely cluster (called the *primary cluster*). SaTScan calculates and reports a logarithm of the likelihood ratio (LLR) for each cluster. As described in the SaTScan User Guide, to evaluate the statistical significance of the *primary cluster*, "a large number of random replications of the data set are generated under the null hypothesis. The p-value is obtained through Monte Carlo hypothesis testing, by comparing the rank of the maximum likelihood from the real data set with the maximum likelihoods from the random data sets. If this rank is R, then the p-value = R/(1 + # simulations)" [[11], p16]. In SaTScan, the default value for the number of simulations is 999.

SaTScan also identifies *secondary clusters *that have a significantly large likelihood ratio but are not the primary cluster [6]. Many secondary clusters are nearly identical to the primary cluster in geographic position and extent; such secondary clusters are usually of little interest, but serve to remind users that the location and size of detected clusters are only estimates [6]. These secondary clusters occur because slight alteration to the circle radius or relocation of the circle centre to a different, nearby point location (thus adding or removing only a few locations to the circle) changes the likelihood ratio only marginally, especially when the newly included or excluded locations have a small population at risk. However, secondary clusters that have no common geographic area with the primary cluster may be of great interest as they are able to reject the null hypothesis on their own strength [11]; thus, they are significant and potentially meaningful from a research and policy perspective.

### Limitations of the Kulldorff spatial scan statistic and SaTScan

SaTScan has made the spatial scan statistic widely accessible, substantially impacting numerous domains in which spatial clusters are of interest (e.g., crime analysis, epidemiology). However, two issues make using the method and interpreting its results nontrivial: (1) SaTScan lacks cartographic support for understanding the clusters in geographic context and (2) results from the method are sensitive to the selection of scaling parameters, but the system provides no direct support for making these choices. Each issue is discussed below.

First, SaTScan does not provide cartographic support to view the identified clusters, nor a visual interface to explore cluster characteristics. Geographic information about the identified clusters (e.g., the centre location, the cluster radius, data entities included in each cluster) is available only as text. In order to visualize the geographic location and size of the clusters, a user must process the textual output and export it to GIS software (e.g., ArcGIS). This is a time-consuming process and inhibits interactive exploration of multiple parameter configurations for interpretation of the results. Because of this limitation, researchers may choose default parameters or make some other arbitrary choices that do not reflect characteristics of the geographic phenomena. Further, in published examples [12-14], it is typical to see that the studied data (e.g., aggregated disease data) and the SaTScan clusters are depicted on two separate maps, making interpretation of SaTScan clusters relative to the original dataset difficult. In one of the few efforts to address this problem, Boscoe and colleagues propose a single integrated map that combines the likelihood ratio with relative risk [15]. Our proposed methodology builds from this core idea of integrating cluster output with relative risk information and takes advantage of both established and novel interactive geovisualization methods to enhance the understanding of SaTScan results.

Second, it is difficult to determine an optimal setting for SaTScan scaling parameters, as discussed in [12,16]. Confusing and even misleading results are possible if the parameter choices are made arbitrarily. The focus of the research presented here is on the aforementioned maximum-size parameter. The default maximum-size setting of 50% seldom produces usable, informative results because the reported primary cluster often occupies a large proportion of the study area [17]. The task of determining the most appropriate maximum-size is challenging. This choice can be context dependent, influenced both by the geographic scale of processes leading to clusters and by the application goals. In addition, too large of a maximum-size can hide small, homogeneous clusters within larger, heterogeneous ones, and too small of a maximum-size can miss significant, regional-level clusters. SaTScan provides limited guidance for selection of an appropriate maximum-size value. Our research to address this problem prompted a broader question on the sensitivity of SaTScan results to parameter choices.

The sensitivity to the maximum-size parameter has two components. The first is that SaTScan clusters tend to contain heterogeneous contents, particularly when using large maximum-size values. Such clusters are composed of not only the high-risk locations that are of interest in epidemiological cluster detection, but also many low-risk locations that are not of interest. The second component relates to stability of clusters in terms of location and size as the maximum-size value is varied. Adjustments to the maximum-size parameter by only several percentage points may cause large shifts in both the location of identified clusters and their radii. To address these two issues with SaTScan (cartographic output and sensitivity related to both heterogeneous content and circle centre/size instability), we propose a geovisual analytics approach to enhance interpretation of SaTScan results.

### A geovisual analytics approach

Geovisual analytics is a sub-area of the emerging research discipline of visual analytics, with specific focus on problems involving geographic phenomena [18]. Visual analytics is defined in a recent research agenda [[19], p4] as "the science of analytical reasoning facilitated by interactive visual interfaces." Geovisual analytics moves beyond traditional cartographic or GIS output that presents a single, optimal map to the user in support of policymaking. Instead, geovisual analytics allows users to interactively explore visual representations of geographic information, tapping perceptual and cognitive abilities to recognize and process patterns and outliers from a visual scene, link these patterns and outliers to existing mental schemata and knowledge bases, and arrive at an appropriate course of action given the visual input [20,21]. Geovisual analytics can be used to enhance other methods of scientific inquiry to the end of gaining insight about geographic phenomena, drawing conclusions about these phenomena, and directing subsequent scientific investigation or policy action [20]. The research reported here proposes a novel geovisual analytics approach that combines the strength of advanced visualization methods with the analytical capabilities of the spatial scan statistic. The proposed approach is implemented in the *Visual Inquiry Toolkit *(VIT) [22,23], a software package developed at the GeoVISTA Center at The Pennsylvania State University.

We demonstrate the advantages of our approach through the analysis of a cervical cancer mortality dataset for the continental U.S., aggregated by county, from 2000 to 2004. Identification of geographic clusters of increased cervical cancer risk is a particularly important and timely issue in the United States because it is one of the few forms of cancer that is currently preventable. It is believed that 90–95% of the incidence of cervical cancer are caused by the human papillomavirus (HPV) [24-28]. Fortunately, early detection and treatment of cervical dysplasia through regular Pap screening greatly reduces the risk of cervical cancer. Since the introduction of Pap smears in the 1950s, national cervical cancer rates have been reduced by one-half [27]. The recent introduction of the HPV vaccine offers further promise for prevention of cervical cancer [25].

While cervical cancer mortality at the scale of the nation is in decline, geographic disparities remain. It was argued in a 2005 monograph produced by the National Cancer Institute that "Despite the consistent decline in cervical cancer mortality overall," examination of cervical cancer mortality remains essential because "an entrenched geographic pattern of deaths from this disease has persisted for decades" [[28], p1]. The same monograph points to geographic regions in the U.S. that appear to exhibit this pattern; they are in the Deep South, Appalachia, along the Texas-Mexico border, in California, and in the North Plains states. Determination of statistically significant geographic clusters is extremely important for intervention at these locations by physicians and policymakers. Because of the urgency for intervening in areas of high-risk, we focus only upon high-rate clusters; our proposed geovisual analytics method could be applied to low-rate clusters or low- and high-rate clusters together with minor alteration.

In the following Results section, we introduce our novel geovisual analytics methods and discuss how they can be applied to SaTScan results to alleviate the two issues with SaTScan described in the previous session. In the Conclusion section, we summarize the method, describe its limitations, and recommend a procedure for investigating geographic clusters of disease. The final Methods section is reserved for the technical details of data processing, SaTScan application, cartographic mapping, and analysis design.

## Results and discussion

The geovisual analytics methods implemented in VIT directly address the lack of cartographic support for interpreting SaTScan results and the implications of selection of scaling parameters. In our approach, a tabular interface enables loading of multiple SaTScan results without any data manipulation in GIS or other software. This interface is linked dynamically to a choropleth map, a map matrix, and related information visualization tools so that an analyst can select, visualize, and compare SaTScan results produced by different maximum-size values. In the remainder of this section, we describe the sensitivity of SaTScan results to the maximum-size parameter, which is reflected in two ways: (1) heterogeneous contents and (2) instability of clusters in location and size. Although static results from our mapping/visualization tools are used to support this discussion; the tools derive much of their power to support analysis and interpretation from their capabilities for flexible user interaction and dynamic linking among views.

### Heterogeneous and core high risk clusters

When analyzing county-level cervical cancer mortality data for the U.S., we found that SaTScan reports many statistically significant clusters that contain a relatively high proportion of low-risk counties. We describe these regions as *heterogeneous clusters*.

However, we noticed that there are often smaller, homogeneous subsets within heterogeneous clusters that exhibit Standardized Mortality Ratio (SMR) values high enough to reject the null hypothesis on their own strength; other researchers have noted a similar occurrence [14]. We describe these regions as *core clusters*. The phenomenon is caused by the tendency of SaTScan "to identify large areas with large populations but small elevations in risk, since such areas have the highest statistical power. Smaller clusters contained within these areas that have higher elevations in risk but lower, though statistically significant, likelihood ratios, are ignored" [[15], p274]. While SaTScan emphasizes the larger clusters on statistical grounds; the core clusters are of more practical interest from both a research and policy perspective because they represent statistically valid homogenous regions of extreme risk and provide important information at a finer scale. More specifically, as presented here, a core cluster has homogeneous contents, and is stable against parameter variation (e.g., maximum-size). It is either explicitly reported by SaTScan or contained in a larger cluster reported by SaTScan. Although reported as significant clusters by SaTScan, a heterogeneous cluster either has heterogeneous contents or is unstable in terms of location and size under parameter variation. A heterogeneous cluster usually contains one or several core clusters.

**Figure 1:**
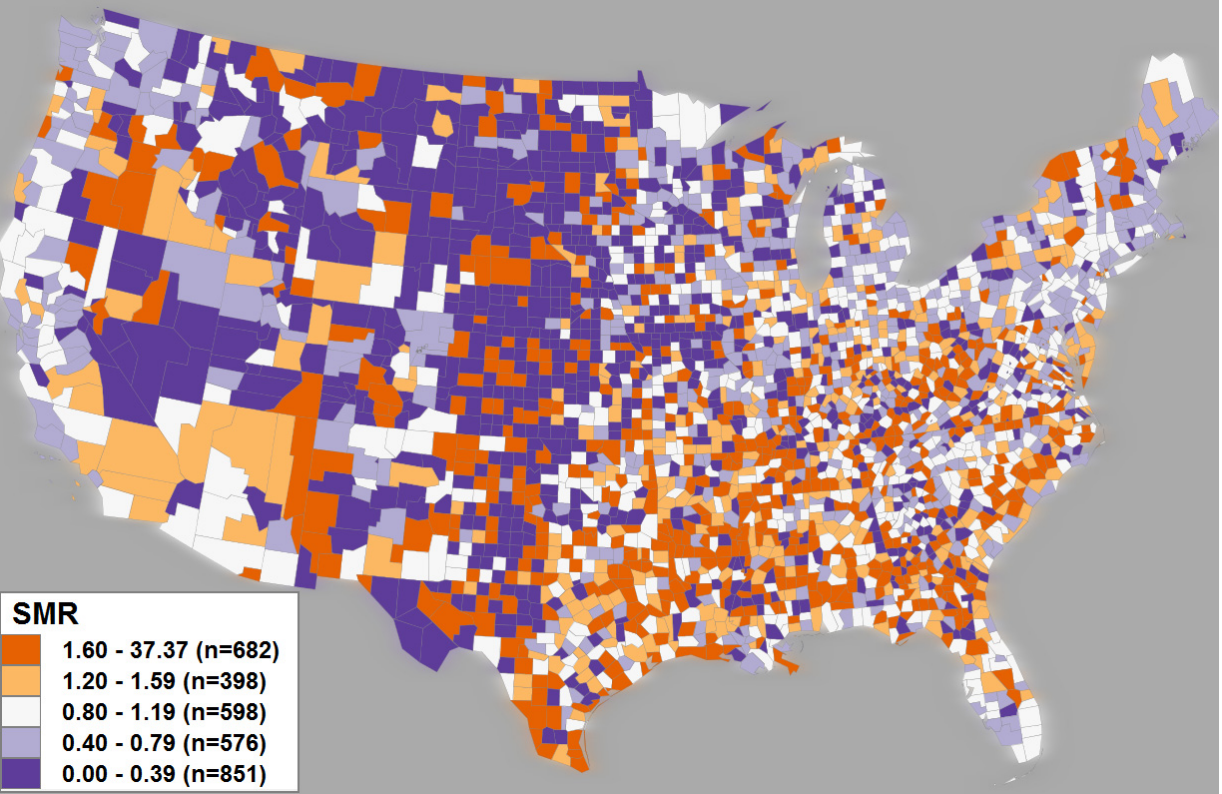
**A choropleth map displaying the SMR for U.S. cervical cancer mortality from 2000–2004**. Cervical cancer SMR is divided into five classes using a modified equal-area classification scheme centred on normal, with breaks at '0.4', '0.8', '1.2', and '1.6'. A blue-orange diverging colour scheme is used: blue representing low-risk, white representing normal risk, and orange representing high-risk. The number of counties belonging to each class is expressed by *n *in the legend.

**Figure 2:**
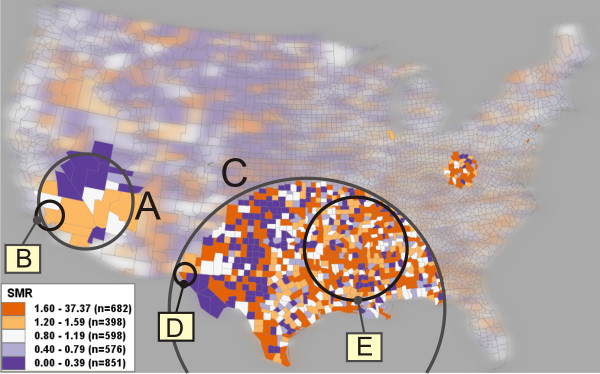
**Heterogeneous versus core clusters**. Clusters A and C are the heterogeneous clusters reported by SaTScan with a large maximum-size. Cluster B, D, E are identified as core clusters by the proposed reliability visualization. The clusters' properties are listed in Table 1. The clusters above are manually circled and labelled for illustration purposes. Other irrelevant clusters (e.g., a single-county cluster near the Great Lakes) are not circled and labelled.

The existence of heterogeneous clusters can be demonstrated by applying SaTScan to the cervical cancer dataset. The SMR map in Figure 1 provides an overview of SMR values by county. Figure 2 displays only those counties contained in clusters reported by SaTScan when the maximum-size parameter is set to 40%. Two heterogeneous clusters are present in Figure 2: clusters A and C. Although these identified regions have statistically higher mean SMR values than the rest of the country, many counties within each cluster exhibit low SMR values (shown in dark blue). Further, it appears as though there are several homogeneous subsets within Cluster A and C that are possibly core clusters (e.g., sub-region E in cluster C and subregion in orange in cluster A). To evaluate heterogeneity of the clusters, we measured the percentage of the total number of counties in a cluster that are not in high risk (i.e., SMR less or equal to '1'); this percentage is subsequently referred to as Pct_NH. Cluster A has a Pct_NH value of 52.4%, and Cluster C has the value of 40.8%. From a practical perspective (e.g., disease prevention or policy making), reporting high risk clusters like A and C that contain a large portion of non-high-risk locations has obvious negative implications if cluster heterogeneity is not recognized.

To facilitate identification of core clusters and avoid potentially misleading heterogeneous clusters, it is necessary to avoid selection of an excessive maximum-size value. For example, the core cluster B (consisting of only Los Angeles County, CA but containing 3.4% of the US population at risk) in Figure 2 is reported as a significant cluster when the maximum-size value is set to 4%. However, as the maximum-size is increased to more than 6%, the core cluster expands to the extent of cluster A. Although cluster A in Figure 2 is statistically valid at a maximum-size above 6%, it is a heterogeneous cluster because 52.4% of its counties (eleven out of twenty-one counties) are not in high risk. Specifically, nine of the eleven counties showing no high risk in cluster A (in purple) have an SMR value of '0.0' (could be extremely low disease risk) and a population value ranging from '404' to '9,263' (extremely low population at risk); the other two counties have SMR less than 1. A SMR value of '0.0' means that there are no case reported in the area, which could be because the risk is low or the population is low. This example demonstrates that a core cluster may be a single county (i.e., cluster B) as long as its SMR and population at risk are sufficiently larger than surrounding areas. Similarly, we suggest that the heterogeneous cluster C contains core clusters D and E. Cluster E is reported when the maximum-size is set at 4% or 6%. Cluster E is more homogeneous than Cluster C because the former has a considerably smaller Pct_NH value than the latter (as shown in Table 1). Identification of core cluster D will be described in the section below on visual identification of core clusters.

**Table 1:** Properties of the clusters discussed in this paper.

Cluster	Pop	Pct_Pop	p-value	SMR	Pct_NH	County#
**A**	8387.8 k	5.76%	0.001	1.25	52.4%	21
**B**	4928.8 k	3.38%	0.001	1.3	0%	1
**C**	23,249 k	16.0%	0.001	1.28	40.8%	790
**D**	360.5 k	0.25%	0.000	1.84	0%	1
**E**	4957.2 k	3.41%	0.001	1.53	30%	260
**F**	878.8 k	0.6%	0.001	1.59	25.5%	47
**G**	454.4 k	0.3%	0.005	1.74	18.8%	16
**H**	60154.3 k	41.3%	0.001	1.14	45.4%	1644

The table list following properties for the clusters: Pop (population at risk), Pct_Pop (the percentage of total population at risk), p-value, SMR (Standardized Mortality Ratio), Pct_NH (the percentage of the counties in a cluster that are not in high risk), and County# (the number of counties contained in a cluster). Extremely small p-values (e.g., less than 0.001) are reported by SaTScan as 0.000, which means extremely small values rather than 0.

On the other hand, when the maximum-size parameter is too small, SaTScan may report only the smallest core clusters, missing regional-level core clusters of homogenous high-risk contents. This occurs when a core cluster has a percentage of the total population at risk above the maximum-size value. For example, we ran scans with a maximum-size of 1%, 2%, and 3%. No cluster is reported in the area where cluster A occupies. This finding suggests that cluster B is the only core cluster in cluster A. We also examined the LLR values of clusters A and B; a LLR value provides a cluster the statistical power to reject the null hypothesis. With a maximum-size of 6%, Cluster B has an LLR value of '25.30' (with the associated p-value of 0.001). The value is much higher than other significant clusters (e.g., one with LLR of '13.77', with p-value of 0.005) and is close to the LLR of cluster A ('31.29', with p-value of 0.001). It suggests that the statistical power of cluster A is contributed primarily by core cluster B.

### Instability of SaTScan clusters

**Figure 3:**
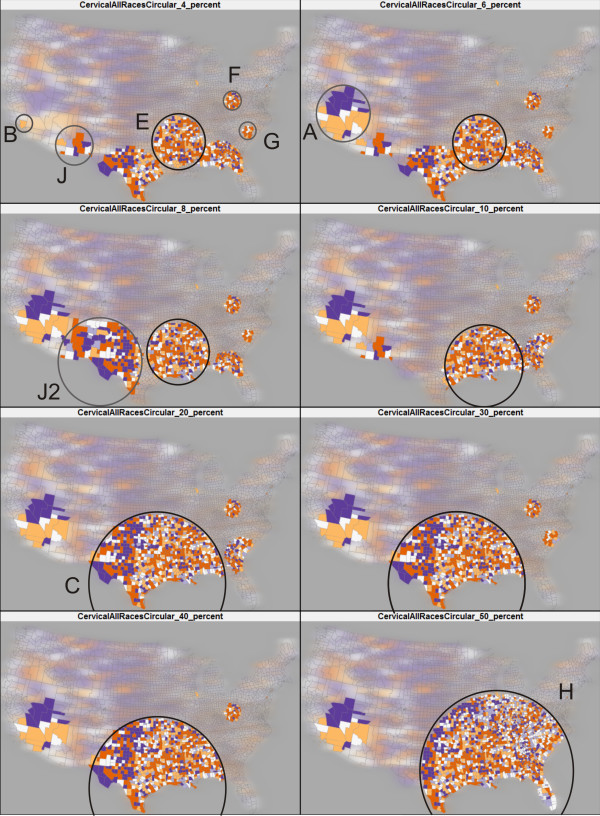
**A map matrix displaying eight SaTScan runs for cervical cancer**. The eight runs increase the maximum-size parameter systematically (4%, 6%, 8%, 10%, 20%, 30%, 40%, and 50%). As the maximum-size parameter is increased, the clusters reported by SaTScan vary in both location and size (as illustrated by the growing black circles and gray circles). For illustration purposes, we carefully draw circles around the boundary of the clusters. For each result, the primary cluster is in a black circle; and some secondary clusters are in gray circles.

In order to obtain core clusters, it is necessary to run SaTScan multiple times using different maximum-size values, particularly for relatively large datasets (e.g., U.S. data aggregated by county). To examine this issue, we ran fifty scans, increasing the maximum-size parameter in 1 percentage point increments from 1% to the default 50%. We then interactively compared the fifty SaTScan runs using the *map matrix *[29] component of VIT. In a map matrix, each SaTScan result is shown on a small choropleth map, allowing for side-by-side comparison; this display technique is also referred to as small multiples. Eight of these scans (4%, 6%, 8%, 10%, 20%, 30%, 40%, and 50%) are displayed in Figure 3 to illustrate the variation in SaTScan results caused by increasing the maximum-size parameter. It can be seen in Figure 3 that, as the maximum-size is increased, the primary SaTScan cluster (marked in a black circle on each small map) expands from the core cluster at a maximum-size of 4% (in circle E) to its fullest extent at a maximum-size of 50% (in circle H). In the process, core clusters F and G, which were not near core cluster E at a maximum-size of 4%, are encompassed by the large heterogeneous cluster H. As shown in Table 1, the contents of the core, primary clusters at 4% are much more homogenous than those in the heterogeneous, primary cluster at 50% (i.e., cluster H). Thus, it appears as though the simple solution to avoid heterogeneity is to restrict the maximum-size.

However, we noticed that as we reduced the maximum-size, the location and size of the core clusters varied significantly, producing a new problem of core cluster instability in both location and size. For example, cluster J is reported at the maximum-sizes of 4%, 6% and 10%; however, it expands into cluster J2 at a maximum-size of 8% and then disappears when the maximum-size is above 10%. Such a finding suggests that a unique maximum-size value might be necessary for identifying the correct bounds of each core cluster, and that the clusters are scale-dependent. Hence, it is unlikely that there is a single, optimal maximum-size value for the entire study extent when scanning a large spatial dataset (e.g., U.S. data aggregated by county). Therefore, we recommend running multiple scans, systematically increasing the maximum-size parameter with each run. The above issue of core cluster instability still raises several important questions: First, how reliable are the clusters reported by a single scan, as compared to those by multiple scans? Second, how do we cope with the sensitivity of SaTScan results to the maximum-size parameter? Lastly, can we identify more stable, homogeneous clusters by running multiple scans, and how? We address these questions in the following sections.

### Visualizing Reliability of SaTScan clusters

To assist discrimination of stable, core clusters from heterogeneous and/or unstable ones, we have developed a method that we term *reliability visualization*. The method visualizes the reliability that a county is reported within a cluster when SaTScan is run multiple times with a systematically varying maximum-size parameter. In epidemiology, reliability is defined as the capacity of a test to give the same result – positive or negative – on repeated applications [30]. Reliability is separated into two different types: (1) intra-observer reliability (agreement of results from the same diagnostic test being given at multiple times or with multiple settings) and (2) inter-observer reliability (agreement of multiple observers on the result of one diagnostic test). We consider reliability visualization to be of the intra-observer type of reliability because we are considering the agreement of results from multiple SaTScan runs, each with a slightly different parameter configuration. Reliability is estimated by Equation 2:

(2) $$R_{i}=\frac{C_{i}}{S}$$

where *R*_*i *_is the reliability value for location *i, S *is the total number of scans, and *C*_*i *_is the number of scans for which that location *i *is within a significant cluster. The reliability measure has a value range from '0' to '1,' where '0' means that the location is not found in a significant cluster in any of the scans and '1' means that the location is within a significant cluster in all scans. The reliability score measures the stability of clusters reported by multiple scans. Reliability is distinct from the concept of validity, which is a measure of the probability that the cluster represents a true high-risk region. Therefore, the goal of reliability visualization is to identify stable core clusters rather than to evaluate the validity of the core clusters. Since we are applying it to the results of an analysis that measures validity, the end result is to identify the locations that are reliable within a significant high risk cluster.

**Figure 4:**
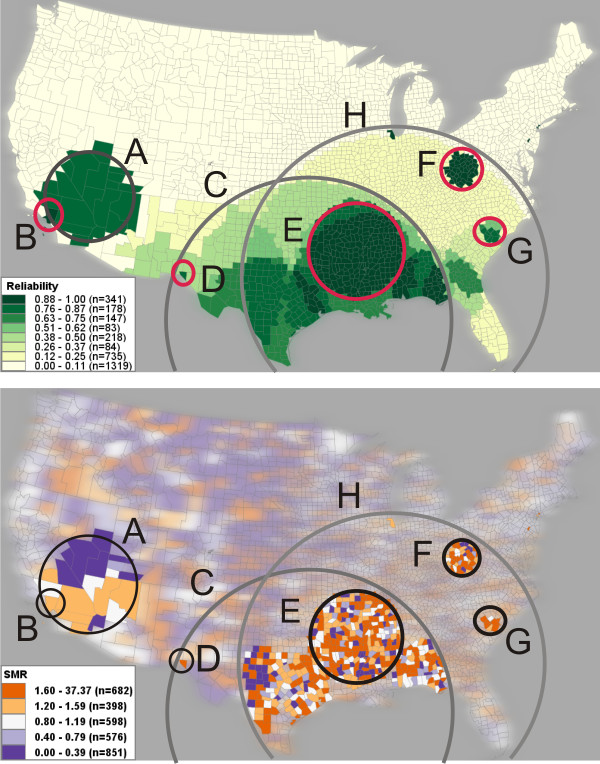
**Visualization of reliability and SMR**. Top: A reliability visualization of the eight SaTScan runs mapped in Figure 3. The reliability values are classified into eight categories (one category for each possible value); darker shading represents a higher reliability. Boundaries of the heterogeneous clusters (A, C, and H) and the core clusters (B, D, E, F, and G) marked in Figure 3 are also marked here for comparison. Bottom: A choropleth map of SMR filtered to show only those counties with a reliability score above '0.875' (the top two classes in Figure 4, top). Boundaries of the core clusters are marked. The clusters above are manually circled and labelled for illustration purposes.

Using VIT, reliability scores for each county were calculated using the eight scans in Figure 3 and are visualized in Figure 4 (top), outlining the same five clusters (i.e., cluster A, B, C, D, and E) from Figure 2. The map of SMR (Figure 4, bottom) has been filtered to show only those counties with a reliability score above '0.75', the top two classes in the legend of Figure 4 (top).

### Visual identification of core clusters

Interpreted with the SMR map, the reliability visualization facilitates initial visual discrimination of core clusters from heterogeneous ones. The task involves two steps: (1) interpreting the reliability scores with SMR values and (2) comparing the reliability score of a cluster with the places neighbouring or inside the cluster.

When interpreting the reliability visualization in tandem with SMR values (step #1 above), a heterogeneous cluster can be identified if it exhibits heterogeneity in both reliability scores and SMR values. Examples are clusters C and H in Figure 4 (top), which are reported during the scans of 40% and 50% respectively. In contrast, a cluster that contains homogeneous, high reliability scores and high SMR values is likely to be a core cluster. For example, the reliability visualization (Figure 4, top) displays three potential core clusters: E (centred upon the lower Mississippi River basin), F (centred upon West Virginia), and G (centred upon South Carolina). By comparing the reliability visualization with the SMR map (Figure 4, bottom), we found the three clusters are significant high risk regions (all have SMR > '1.2' and p-value < '0.05') and are found to be reliable (all counties have reliability scores above '0.75'). In addition, the heterogeneity measure (Pct_NH) for clusters E, F, and G are significantly less than clusters C and H (as shown in Table 1). Therefore, we can reasonably conclude that clusters E, F, and G are reliable core clusters. The reliability visualization also shows some single county clusters (e.g., Cook County, Illinois containing the city of Chicago) that are linked to no obvious heterogeneous clusters and are repeatedly reported by SaTScan with different maximum-size values.

When interpreting the reliability score, an analyst must compare both sub-regions of high risk within the cluster to adjacent areas within the cluster (i.e., an internal comparison) and the cluster as a whole to adjacent areas out of the cluster (i.e., an external comparison). This is particularly important for the clusters that have high a heterogeneity measure (Pct_NH). For example, cluster A in Figure 4 has homogenous reliability scores of '0.875' or higher, therefore it initially appears to be a core cluster. However, cluster B, an internal sub-region of cluster A, has a higher reliability score of '1' and is reported as a single county cluster by the 4% SaTScan run. The comparison of reliability scores and SMR values between cluster A and B suggests that cluster A is possibly a heterogeneous cluster dependent upon core cluster B. The comparison can also be conducted between a cluster and its outside adjacent places. For example in Figure 4, cluster D, containing only a single county, has a reliability score of '0.875' and has an SMR of '1.84'. Cluster D is likely a core cluster because it is surrounded by counties with very low reliability scores. Meanwhile, cluster J (and J2) in Figure 3 is not stable as the maximum-size varies and is therefore probably a heterogeneous cluster.

### Evaluating the reliability visualization

**Figure 5:**
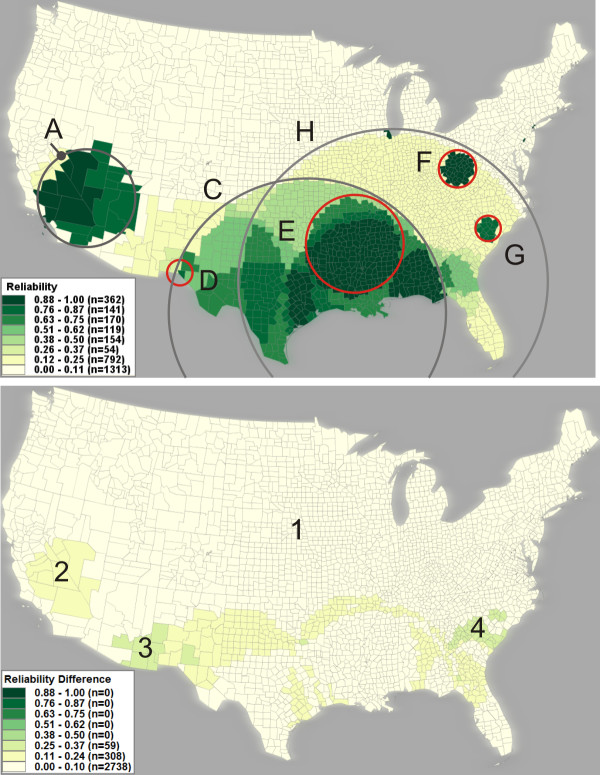
**Evaluate reliability visualization by comparing reliability maps**. Top: a reliability map with another set of eight SaTScan runs (5%, 7%, 9%, 11%, 19%, 29%, 39%, 49%). This set of eight runs offer approximately same scales at the previous eight runs (4%, 6%, 8%, 10%, 20%, 30%, 40%, and 50%) that produces the reliability visualization in Figure 4. For illustration purposes, we carefully draw circles (A, C, D, E, F, G, H) that approximately match the corresponding clusters in Figure 4. As compared to Figure 4, the location and size of most core clusters (in dark green) remain relatively stable in Figure 5(top). Bottom: a map that shows the different in reliability score between the Figure 5(top) and Figure 4(top). Areas in light yellow (e.g., marked as #1) show no difference in reliability score. Areas in yellow (e.g., #2) show a difference of '0.125', and areas in light green (e.g., #3 and #4) show a difference of '0.25'.

To evaluate whether the reliability visualization technique is consistent enough to help users understand and cope with the sensitivity of SaTScan results to the choices of maximum-size, we generated another reliability visualization (Figure 5) based on a different set of eight maximum-size values: 5%, 7%, 9%, 11%, 19%, 29%, 39%, 49%. The set is selected systematically to have the same number of runs and similar maximum-size values as the previous set. By comparing Figure 4 and Figure 5, we found that the location and size of most core clusters (e.g., D, E, F, and G) remain relatively stable. To evaluate the similarity between the two reliability maps, we compared the reliability scores of the two maps. For each reliability map, let vector R = {r_1_, r_2_, ..., r_n_}, where r_*i *_represents the reliability score of county *i *in the map, and county *i *in one map matches the county *i *in the other map. The similarity between the two vectors R1 and R2 is measured in terms of Euclidean distance [31]; and the similarity value ranges between '0.0' (completely different) and '1.0' (completely identical). The similarity value of '0.931' for the two vectors indicates a high similarity between the two reliability maps. Figure 5 (bottom) visually shows the differences in reliability score between the two reliability maps. The differences are slight (ranging from '0.125' to '0.25' in reliability score) and most of them occur at regions with low reliability scores; therefore, the differences in reliability score found between the two maps barely affect the identification of reliable clusters. In summary, the comparison suggests that the reliability visualization, when applied to multiple SaTScan results produced by systematically chosen maximum-sizes, can effectively reduce the influence of parameter choices on the interpretation of SaTScan results.

**Figure 6:**
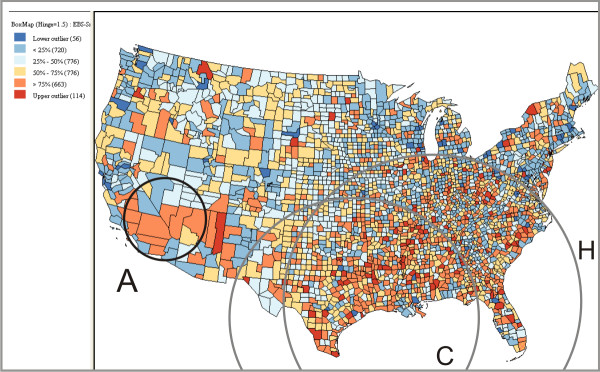
**Smoothed SMR produced by the Empirical Bayes Smoothing method available in GeoDa**. For illustration purpose, we draw three circles (A, C, H) that approximately match the cluster A, C and H in Figure 4. The counties with top 25% high SMR (in red and dark orange) are considered in high risk.

**Figure 7:**
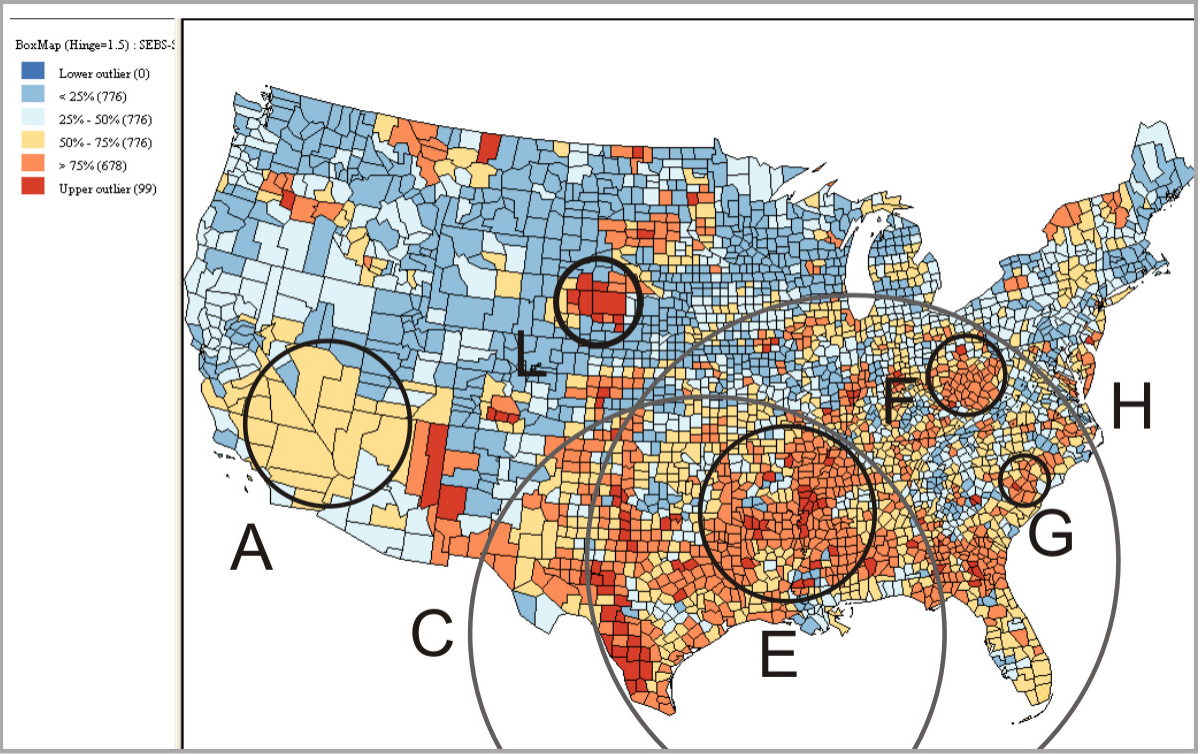
**Smoothed SMR produced by the Kafadar spatial smoother available in GeoDa**. Because GeoDa does not allow for flexible classification in the same way as VIT, we chose a classification similar to the one adopted in the Figure 1. We consider the counties with top 25% high SMR (in red and dark orange) as high risk. A sparsely-populated region in red (indicates "high risk") is highlighted in the circle L. For illustration purposes, we carefully draw circles (A, C, E, F, G, and H) that approximately match the corresponding clusters in Figure 4.

For further evaluation, we compared our reliability visualization with the maps generated by the Empirical Bayes Smoothing method (Figure 6) and the Kafadar spatial smoother [32] (Figure 7). The comparisons yielded many similarities as well as some differences. The Empirical Bayes Smoothing method produced a similar outcome to the reliability visualization – both methods report high risk regions in Southeastern U.S. and Southern California (cluster A in Figure 7). However, areas of clustering on the Empirical Bayes Smoothing map were much less distinct than those on the reliability visualization, making it difficult to determine significant high risk regions on the Empirical Bayes Smoothing map. Comparison of the reliability visualization to the Kafadar spatial smoother output showed more compelling results. Both methods reported a high risk region in Southern U.S. (cluster C in Figure 7), the Mississippi River basin (cluster E), West Virginia (cluster F), and South Carolina (cluster G). However, the Kafadar spatial smoother reported the Texas-Mexican border as a region of extreme high risk, while the reliability visualization showed this region as less stable than other core clusters. This is a region that SaTScan may have missed due to use of circular scans rather than elliptical scans. The Kafadar spatial smoother also reported Southern California (cluster A) as a relatively low or normal risk cluster and did not identify core cluster B. Furthermore, the Kafadar spatial smoother identified some sparsely-populated regions (e.g., region L in Figure 7) as high risk, which SaTScan does not report.

There are several limitations to Empirical Bayes Smoothing and the Kafadar spatial smoother that may be causing the discrepancies with the reliability visualization. First, through smoothing, isolated groupings of high rate values are averaged into the surrounding low rate values, missing the smaller core clusters (e.g., cluster B, D). Second, although the method smoothes the rates in small population areas, it does not eliminate the small number problem. For example, the smoothed map suggests a high risk region that has sparse population (i.e., region L in Figure 7, which has average population of 3,800). In contrast, SaTScan does not report the region as high risk. Finally, the primary goals and advantages of smoothing methods are to stabilize rates and reduce noise [33] rather than to identify clusters. Although smoothing methods can be used to produce disease maps that dampen the noise in the original data (e.g., for evaluating other methods), clustering methods are still needed to identify and verify clusters.

In summary, the reliability visualization helps users cope with the parameter sensitivity of the Kulldorff's spatial scan statistic by providing one summary view of multiple SaTScan runs completed with varying maximum-size values. The reliability visualization can effectively discriminate core clusters from heterogeneous ones – as demonstrated above. The discrimination is important for (1) identification of spatially small, yet important and stable high risk core clusters (e.g., cluster B, D, E) that can otherwise be easily hidden in the reported SaTScan clusters and (2) for improving the understanding of heterogeneous clusters and their formation. Such discrimination is difficult when interpreting a single scan using traditional visualization methods. The reliability visualization method can be used in any situation where a method's parameter sensitivity is a concern and when analysis of multiple outcomes is needed, thus allowing application beyond the maximum-size parameter of the Kulldorff spatial scan statistic.

## Conclusion

The Kulldorff spatial scan statistic and the SaTScan software represent an important first step to cluster identification and interpretation. However, two issues make using the method and interpreting its results non-trivial: (1) SaTScan lacks cartographic support for understanding the clusters in geographic context, and (2) results from the method are sensitive to the selection of scaling parameters. The system provides neither direct support for making these choices, nor methods to evaluate the results from selection of various parameters.

**Figure 8:**
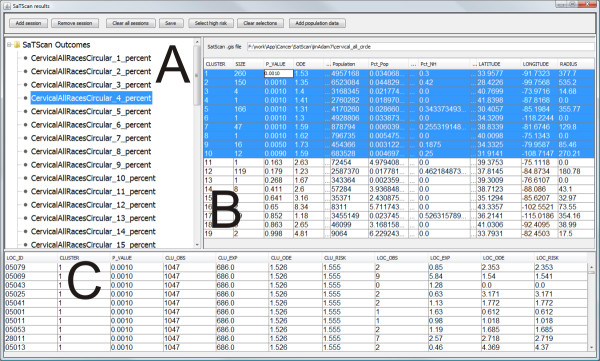
**A tabular user interfaces to display and interact with multiple SaTScan output files**. VIT loads SaTScan output in text format. Display panel A shows results for the fifty SaTScan runs; selection of a specific file or set of files displays clusters identified in those runs in tables B and C as well as in the linked choropleth and parallel-coordinate plot views. The result with maximum-size of 4% is currently selected and highlighted. Table B displays clusters reported by SaTScan, with significant clusters highlighted. Table C displays counties contained within those clusters displayed in Table B.

The VIT addresses the first issue by providing a tabular interface (Figure 8) for loading and visualizing identified cluster footprints on a geographic map, without requiring prior data manipulation in GIS software. For the second issue, we contend that it is unlikely that there is one optimal maximum-size value for the entire study extent when scanning a relatively large spatial dataset (e.g., U.S. data aggregated by county). Rather, it is probable that the core clusters exist at different geographic scales, each requiring a unique maximum-size value for detection. Therefore, we recommend completing multiple SaTScan runs, each adjusting the maximum-size value systematically. The map matrix (Figure 3), reliability visualization (Figure 4), and interactive tabular interface (Figure 8) can then be used to for interactive investigation and interpretation of the multiple scans. In summary, the proposed geovisual analytics approach compliments statistical approaches in cluster identification, enhancing the interpretation of identified clusters.

It is important to note that our geovisual analytics methods, as they are currently proposed, have limitations. A primary limitation is that the methods are contingent upon the quality of the incoming SaTScan results. It is possible that the incoming SaTScan results can be flawed themselves due to limited user guidance, arbitrary parameter selections, the miss-match between any geometric shape scan (whether circular and elliptical) and some real-world geographic clusters, or general misuse of the software. Because of this, we recommend comparison of SaTScan outcomes with other, independent clustering techniques (e.g., methods provided by WinBUGS, ClusterSeer, etc.). At present, VIT is not equipped with sufficient methods (e.g., rate-smoothing methods) for appropriate comparisons. Secondly, visual interpretation can be more difficult than statistical interpretation, particularly when the analyst is not accustomed to using visual methods of analysis. Therefore, it is extremely important that visual evidence is used to complement statistical evidence, and not to replace it. Further, we recommend consultation with multiple epidemiologists and other domain experts prior to the development and implementation of health care policy.

**Figure 9:**
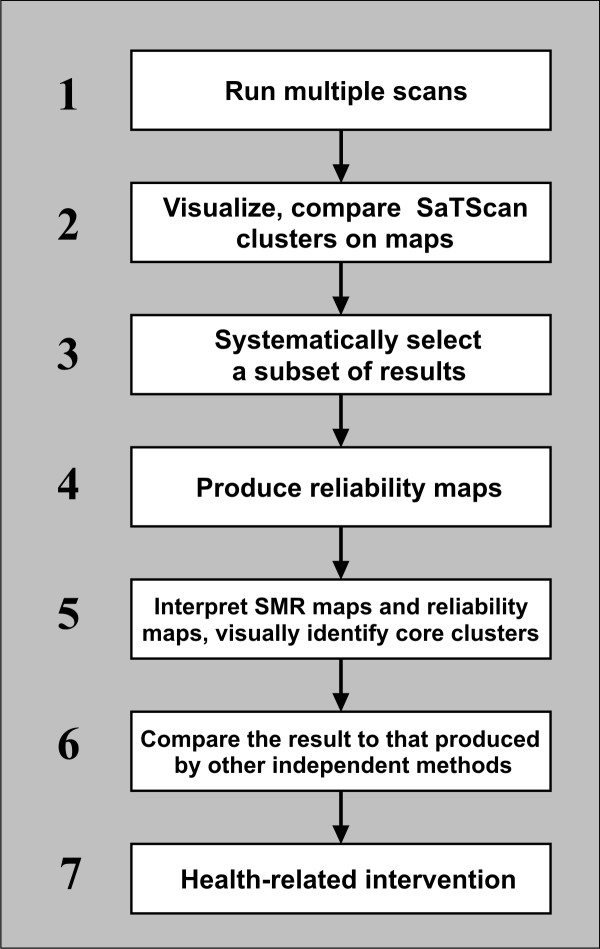
**A logic process for geovisual analytics of SaTScan results**. We propose this seven-step process for addressing the sensitivity issues of SaTScan, and enhancing the interpretation of SaTScan result.

Figure 9 summarizes our suggested procedure for interpreting SaTScan results. First, SaTScan should be run multiple times, starting from a small maximum-size (e.g., a default 1% or the percentage of total, nationwide population at risk contained by the most populated county) and increasing to the 50% default value. Second, the SaTScan results should be visualized in a map matrix for side-by-side comparison of different maximum-sizes. Third, the map matrix should be used to select 6–10 SaTScan runs that are representative of the maximize-size parameter range. Fourth, the reliability visualization should be constructed based on the selected SaTScan runs. Fifth, core clusters should be discriminated from heterogeneous clusters through interpretation of the reliability visualization and the SMR map. Sixth, the interpretation of core clusters should be confirmed by comparing the results to other independent techniques and consultation with domain experts. Finally, and most importantly, the appropriate health care policy should be developed for intervention in core clusters of increased disease risk.

## Methods

### Cervical cancer data processing and SaTScan runs

The cervical cancer mortality data for the United States between 2000 and 2004 were obtained from the National Cancer Institute using the Surveillance, Epidemiology, and End Results (SEER) program via the SEER*Stat software. The data are aggregated into 3,105 county and county-like enumeration units in the forty-eight contiguous states of the U.S., including the District of Columbia. We removed Alaska and Hawaii to ensure geographic continuity for the cluster analysis. The mortality data were originally grouped into nineteen age groups by county of residence at the time of death; the first two age groupings (age 0 and age 1–4) were combined to produce eighteen 5-year intervals through age 85. The U.S. female population, estimated by the U.S. Census for each year in the study period, was summed to produce the population at risk.

SaTScan evaluates disease risk in terms of relative risk, reporting the ratio of observed to expected cases for both the total cluster and individual enumeration units within the cluster. This ratio, when applied to mortality rates, is formally known as the Standardized Mortality Ratio (SMR) in epidemiology, expressed as the ratio of observed to expected deaths. An SMR of '1' suggests no difference in risk, lower than '1' suggests low-risk, and larger than '1' suggests high-risk. To calculate SMR, we obtained age-specific observed deaths from SEER*Stat. We calculated the number of expected deaths using Equation 3:

(3) $$\sum_{n}p_{i}*\frac{C_{i}}{P_{i}}$$

where the population is categorized in *n *age groups, *p*_*i *_is the population of a county in an age group, *C*_*i *_is the sum of the mortality count for all the counties in the age group, *P*_*i *_is the sum of the population of all counties in the age group.

We chose to visualize indirectly adjusted relative risk rather than a directly adjusted mortality rate because it is less subject to bias than the directly adjusted rate in areas with small populations. Furthermore, SaTScan evaluates and reports clusters in terms of relative risk, which is comparable to SMR. Therefore it is easier to analyze SaTScan results by visualizing SMR.

### Visualizing aggregated disease data with a choropleth map

The choropleth mapping technique is commonly applied to aggregated disease data and Brewer and Pickle contend it is well suited to this application [34]. Choropleth maps symbolize numerical attribute values for each enumeration unit in the study area by filling the area with a colour or shade that represents the value, allowing for visual discrimination of spatial clustering based on the adjacency of similar colours [35]. Because of their ease in creation and familiarity with the general public, the cartographic literature pays a great deal of attention to the limitations of the choropleth technique and the possible map reading errors these limitations may cause. We describe the recommended visualization solutions that address three important issues that must be considered when representing disease data in choropleth maps: (1) data classification, (2) colour selection, and (3) the small number problem. This discussion serves as an introduction for those not familiar with the cartographic techniques already developed for visualizing aggregated disease data and acts as a segue into the description of our methods specific to enhancing SaTScan outcomes with geovisual analytics.

The first concern with choropleth mapping is the classification strategy imposed on the dataset. There are two forms of choropleth maps: (1) classed and (2) unclassed. Classed choropleth maps divide the attribute space into a set of intervals, filling all enumeration units in the same interval with a single colour, while unclassed or n-classed choropleth maps mathematically relate each attribute value to a position on a colour ramp, pairing every unique value with a unique colour [34]. We chose the classed choropleth map (as opposed to the unclassed map) for visualizing the disease risk because of its simpler interpretation and its emphasis on the hot spots of low- and high-risk [36]. Like any abstraction, data classification has the potential to mislead the analyst [37]. Thus, care must be taken to select a classification approach that fits the data and the application. The two most important parameter settings for choropleth classification are the number of classes into which the data are divided and the method of classification. Depending on the map complexity, most authors agree that the appropriate number of classes for choropleth mapping ranges from five to seven [34,38]. Application of fewer total classes results in a map pattern that is heavily dependent on the position of class breaks, and therefore unstable; application of more total classes (or use of an unclassed choropleth map) makes it more difficult to comprehend general map patterns and retrieve specific information from the resulting map. For analysis of SMR in relation to spatial cluster results, we recommend a five-class, modified equal-interval classification scheme centred on normal for mapping cervical cancer SMR by US county, with breaks at '0.4', '0.8', '1.2', and '1.6'. Expert input from epidemiologists was used to select meaningful SMR class breaks influential to policymaking (e.g., '0.4' and below representing extreme low-risk, '1' representing normal conditions, and '1.6' and above representing extreme high-risk). Similar classifications are used in other research involving the visualization of SaTScan results [12,15,39].

The second concern with choropleth mapping is the choice of colour scheme used to symbolize the determined classes. Improper colour selection inhibits map users from retrieving both general patterns and specific details from the map display [40]. SMR is a bipolar dataset centred upon '1' with increasing low- and high-risk towards either antipode. Because of this, a divergent colour scheme (i.e., ordered colour steps in two directions away from a middle point [41]) is recommended for choropleth representation of SMR. Specifically, we used a blue-orange diverging colour scheme, with high-risk in dark orange to draw attention, normal risk in white, and low-risk symbolized in dark blue (this is a colour combination usable by those with common colour deficiencies). Figure 1 presents a five-class choropleth map of cervical cancer SMR by US county using the aforementioned classification and colour scheme.

The third concern with choropleth mapping is the small numbers problem. Disease rates tend to be unstable for enumeration units that have a small population at risk or for diseases that are rare [2]. For such enumeration units, the inclusion or removal of only a few cases may have a large influence on the rate itself. Because disease rates vary widely for enumeration units with the small unit problem, epidemiologists have less confidence in the rates when developing a public health policy. Cervical cancer is unfortunately frequent enough to be outside the category of a rare disease, but still infrequent enough to exhibit the small numbers problem when aggregated to the county level. A solution for the problem is spatial smoothing. The basic idea is to borrow information from neighbouring regions to produce a more stable and less noisy estimate of disease rates for each enumeration unit, thus separating out spatial pattern from noise [33]. One representative smoothing method, Empirical Bayes Smoothing, adjusts (or smoothes) rates upward or downward, pulling them toward the national or regional average according to the population on which they are based [42-44]. Consequentially, the rates for small areas are adjusted more than those for large areas [2]. However, the general Empirical Bayes Smoothing method involves no geography and does not perform well in the high noise situations typical of epidemiologic data [45]. Kafadar [32] proposed a more accurate smoother that was developed specifically for geographic data; we refer to it as the Kafadar spatial smoother. For comparison purposes, this research adopted both smoothing methods, which are provided in GeoDa [46]. The smoothed SMR produced by Empirical Bayes Smoothing and the Kafadar spatial smoother are shown in Figure 6 and Figure 7 respectively.

Finally, choropleth mapping suffers problem visual bias (e.g., some high-risk, densely-populated, but geographically tiny regions are often visually hidden). An area-based cartogram [35,47] would be useful to address the problem, and this is one of our future research works.

### Analysis design

A primary objective of this research was to determine the sensitivity of SaTScan results to the maximum-size parameter. It is important to reiterate that the maximum-size parameter can be either the percentage of the total population at risk or the geographic size of the circle. We use the former because it is the default setting in SaTScan. To understand the sensitivity of SaTScan results to the maximum-size parameter, we ran the SaTScan spatial scan statistic on the cervical cancer dataset fifty times, starting with a maximum-size of 1% and increasing the parameter by an interval of 1 percentage point with each run until reaching the default value maximum-size value of 50%. For each run, we set the number of Monte Carlo replications to 999. The SaTScan software allows users to set the maximum-size in two ways: (1) through restriction of the upper limits of the maximum-size parameter before running the scan or (2) through retention of the default maximum-size of 50%, but selecting for the software to report only circles with a maximum-size value below the desired value. We adopted the second method to avoid pre-selection bias, as recommended by Kulldorff [11]. The output for each SaTScan run produces a place ID for each identified cluster; the FIPS code was used as the place ID for this research. The FIPS code at the county level is a five-digit textual code that uniquely identifies counties and county equivalents in the United States. The user can load the files of the fifty SaTScan results into VIT as a batch by specifying the folder where the files are stored in the computer. They are examined in tabular form using the interactive user interface shown in Figure 8. Once the results have been loaded into the VIT's tabular interface, an analyst can interactively investigate each cluster or the individual counties. By selecting one or multiple cluster(s) in the tabular interface, the analyst can select and highlight the cluster(s) on the choropleth map, blurring those counties not included in the selection. We drew a circle around the boundary of each cluster for illustration purposes only; this feature is not implemented in VIT. Specific details (e.g., SMR, p-value, population, etc) about each cluster and individual counties can be interactively retrieved from the map and the tabular interface.

## Abbreviations

FIPS code: Federal Information Processing Standards code; HPV: human papillomavirus; LLR: logarithm of the likelihood ratio; maximum-size: the maximum radius size of a scan circle; Pop: the population at risk of a cluster; Pct_NH: the percentage of the counties in a cluster that are not in high risk; Pct_Pop: the percentage of the total population at risk; SaTScan: Kulldorff's spatial scan statistic and its software implementation; SMR: Standardized Mortality Ratio; VIT: Visual Inquiry Toolkit.

## Competing interests

The authors declare that they have no competing interests.

## Authors' contributions

The study was conceived by EJL and AMM. JC analyzed the spatial scan statistics method and the SaTScan results. JC developed and implemented the novel geovisual analytics methods presented in the text, evaluated the methods, and compared that to the other techniques. The cervical cancer mortality dataset was compiled by AN. SaTScan runs were completed by JC and AN. The visualization of cervical cancer mortality clusters was interpreted by JC, RER, EJL, and AMM. The manuscript was prepared by JC and RER and reviewed by AMM and EJL. EJL assisted in drafting the section on reliability and AN assisted in drafting the section on data. EJL provided epidemiological guidance and AMM provided cartographic and visualization guidance during revisions of the manuscript. All authors read and approved the final manuscript.
